# Investigating Physical Activity as a Predictor of Psychological Distress in UAE Nursing Students

**DOI:** 10.3390/healthcare13172112

**Published:** 2025-08-25

**Authors:** Eman Abdelaziz Ahmed Dabou, Shukri Adam, Mona Gamal Mohamed, Mary Grace Carezon Bedolido, Kim Ashley Militar

**Affiliations:** 1 Adult Health Nursing Department, RAK College of Nursing, RAK Medical and Health Sciences University, Ras Al-Khaimah P.O. Box 11172, United Arab Emirates; eman.abdelaziz@rakmhsu.ac.ae; 2 Adult Health Nursing Department, Nursing College, Alexandria University, Alexandria City 11865, Egypt; 3 Community Health Nursing Department, RAK College of Nursing, RAK Medical and Health Sciences University, Ras Al-Khaimah P.O. Box 11172, United Arab Emirates; shukri@rakmhs.ac.ae; 4 BSN-Student, RAK Medical and Health Sciences University, Ras Al-Khaimah P.O. Box 11172, United Arab Emirates; mary.22904019@rakmhsu.ac.ae (M.G.C.B.); kim.22904042@rakmhsu.ac.ae (K.A.M.)

**Keywords:** physical activity, psychological distress, nursing students, UAE, predictors, mental health, exercise, stress management

## Abstract

**Background:** Psychological distress is one of the leading causes of ill health in the United Arab Emirates (UAE). Nursing students often report higher levels of stress than the general population. Identifying the determinants of mental distress is essential to raise awareness and enable universities to implement preventive interventions. **Aim**: To examine the relationship between physical activity and psychological distress among nursing students at RAK Medical and Health Sciences University. **Methods**: A descriptive cross-sectional design was employed. A total of 187 students completed a three-part survey: (I) sociodemographic characteristics, (II) the International Physical Activity Questionnaire-Short Form (IPAQ-SF), and (III) the Depression Anxiety Stress Scale-21 (DASS-21). **Results**: Among participants, 28.3% were inactive, 36.9% engaged in moderate activity, and 34.8% engaged in vigorous activity. Significant associations were observed between physical activity and gender (χ^2^ = 9.64, *p* < 0.001), nationality (χ^2^ = 8.09, *p* = 0.01), anxiety (FET = 99.34, *p* < 0.001), and stress levels (χ^2^ = 12.41, *p* = 0.05). Regression analysis showed that gender, nationality, anxiety, and stress significantly predicted physical activity levels (F(3,183) = 62.47, *p* < 0.001), explaining 51% of the variance (R^2^ = 0.506, adjusted R^2^ = 0.498). **Conclusion**: Physical activity among nursing students was significantly associated with gender, nationality, anxiety, and stress. Programs that promote physical activity may help reduce psychological distress and improve students’ health and well-being. Failure to address high levels of stress and anxiety may increase the risk of burnout in future professional practice.

## 1. Background

Physical activity (PA) is defined by the World Health Organization (WHO) as any bodily movement produced by skeletal muscles that requires energy expenditure. This involves all movement, including during work, leisure, and transport. This is categorized from inactivity to vigorous on a Likert scale for measurement [1]. Regular PA is proven to help manage and prevent non-communicable diseases (NCDs). It has also been shown to reduce hypertension, maintain a healthy body weight, improve quality of life, and improve well-being by inducing positive mental health [2]. PA, as a concept, however, is confused with individual terms such as physical exercise and sport. Physical activity encompasses exercise and sport and should not be limited to individual definitions. A lack of physical activity is a key factor contributing to academic stress and psychological distress among students [2,3].

Psychological distress is generally defined as a state of emotional suffering, most often related to depression and anxiety, and later expanded to include stress as a core dimension. It is also referred to as mental distress. Early definitions defined psychological distress as a state of emotional suffering consisting of symptoms related to depression and anxiety [4]. However, research focusing on young adults and exploring many different distress-inducing determinants added stress as a further dimension. It was then referred to as a broader manifestation of mental health problems identified through the severity of symptoms of depression, anxiety, and stress [5]. This notion was further elaborated as an umbrella term involving depression, anxiety, and stress by more recent research [6,7].

However, the most appropriate definition used in this study was formulated based on decade-long research, including all three dimensions of stress, anxiety, and depression. Psychological distress is “Any range of symptoms and experiences related to a person’s internal life; feelings of being troubled, confused or out of the ordinary” [8]. This definition is appropriate for this research as it allows the identification of stress, anxiety, and depression while also leaving open the room for identifying any further dimensions that may induce psychological distress [9,10].

Common factors that affected academic stress were identified by Tran et al. 2022 as extensive course loads and lack of physical activity [11]. This resulted in a range of behaviors, such as a change in concentration span, disturbed sleep, irritability, mood swings, and fatigue [12]. This supports the findings of earlier studies that nursing students’ psychological well-being and PA are correlated [13]. Once again, a proportion of the studies reported that gender discrepancy is a key area of interest, as females show higher Stress and anxiety levels than male students [14]. Anxiety and depression are the highest concerns among pharmacy students in Saudi Arabia, affecting 41.6% and 36.4%, respectively. This high prevalence has been shown to interfere with their learning, affect their achievements, and impair their clinical practice. The results of that study showed that high stress and anxiety, and their prevalence, have major implications for the quality of a student’s life [8,15]. The Government in the United Arab Emirates (UAE) is working collaboratively to improve the public health condition in the state, with a special focus on NCDs and mental health [16]. Specific data shows that one of the most common mental health problems in the UAE includes depression associated with anxiety, accounting for around 4–5% of the population [13]. A further report shows that about 75% of psychological conditions across the UAE are associated with depression and anxiety [17]. This notion is important to consider, as anxiety and depression are among the UAE’s top ten causes of ill health. The university period is considered to be a time of evolution and great change for young people. During this stage, individuals first encounter acute and chronic risky situations, which put them at risk for mental health problems. Despite the established benefits of physical activity on mental health, there are few worldwide studies focused on nursing students’ physical activity and psychological problems. Few studies have been conducted in the UAE to assess physical activity levels [18,19]. The current research is the first in Ras Al Khaimah, one of the UAE’s emirates, to focus on nursing students’ physical activity levels and psychological problems. Therefore, this study aimed to determine the relationship between physical activity and psychological distress levels among nursing students.

## 2. Method

### 2.1. Study Design and Setting

A descriptive cross-sectional research design was used to investigate the relationship between physical activity and psychological distress among nursing students in Ras Al-Khaimah, United Arab Emirates. Data was collected from 1 August to the end of November 2023.

### 2.2. Sample Size and Sampling

The study was conducted at RAK Medical and Health Science University, College of Nursing (RAKCON), in the emirate of RAS Al-Khaimah, UAE. The sample was recruited from RAK CON, including the BSN and RN-BSN programs. The accessible and target population included students enrolled in the BSN degree programs (BSN and RN-BSN) at RAKCON. All students were enrolled in the BSN program from year one to year four and in the RN BSN from year one to year two of both genders and aged over 18 years.

Students who had been ill during the month preceding data collection or who declined participation were excluded. The sample size was calculated based on the total number of students in the four BSN years (100, 70, 66, and 74 for BSN years 1, 2, 3, and 4, respectively), for a total of 310 students.

A convenient sampling technique was adopted in this study. This sample size was calculated by using the Roasoft program (2004), assuming a 90% response rate, 95% confidence interval (CI), and a 5% error margin of 5% from 310 total nursing students in RAKCON [20]. The sample size (n) was 187.

### 2.3. Study Measure

The data collection instrument was divided into three parts. **Part I** included the sociodemographic characteristics. **Part II** was the International Physical Activity Questionnaire- Short Form (IPAQ-SF). **Part III** was the Depression Anxiety Stress Scale-21 (DASS-21).

**1. Sociodemographic characteristics** include age, gender, nationality, program, and year of study.

2. International Physical Activity Questionnaire-Short Form (IPAQ-SF).

IPAQ-SF commenced in Geneva in 1998 and was followed by extensive reliability and validity testing undertaken across 12 countries (14 sites) in 2000. The reliability and validity were 0.8 and 0.3, respectively [21]

The short form of IPAQ-SF consists of seven questions assessing the frequency and duration of participation in vigorous, moderate, and walking activities as well as the time spent on these activities. The participants reported the frequency and duration of their vigorous and moderate physical activities and walking per week. The seven questions asked about the time the participants spent physically active in the previous seven days. According to the official guideline criteria, the metabolic equivalent of task (MET) of vigorous activity is 8.0, moderate activity is 4.0, and walking is 3.3; to estimate the total METs [22]. The reliability of the IPAQ-SF was evaluated in the current study. It was excellent (Cronbach’s α = 0.90). Additionally, the content validity was verified in the UAE, and it was found to be valid.

**3. Depression Anxiety Stress Scale-21 (DASS-21).** DASS-21 was a well-established instrument for measuring depression, anxiety, and stress with good reliability and validity reported from Hispanic American, British, and Australian adults [23,24]. The DASS-21 was a set of three self-report scales designed to measure the emotional states of depression, anxiety, and stress. The three DASS-21 scales contained seven items, divided into subscales; the depression scale assessed dysphoria, hopelessness, devaluation of life, self-deprecation, lack of interest/involvement, anhedonia, and inertia. The anxiety scale assessed autonomic arousal, skeletal muscle effects, situational anxiety, and subjective experience of anxious affect. The stress scale is sensitive to levels of chronic non-specific arousal.

It assessed difficulty relaxing, nervous arousal, being easily upset/agitated, being irritable/over-reactive, and being impatient. Scores for depression, anxiety, and stress were calculated by summing the scores for the relevant items. The categories of total score for each dimension of the DASS scale are as follows: for depression, normal (0–9), mild (10–13), moderate (14–20), severe (21–27), and extremely severe (28+); for anxiety as follows: normal (0–7), mild (8–9), moderate (10–14), severe (15–19), and extremely severe (20+); for stress as follows: normal (0–14), mild (15–18), moderate (19–25), severe (26–33), and extremely severe (34+). Internal consistency reliability for the original scale scores (range from 0.82 to 0.97) of the DASS-21 in clinical and nonclinical samples [25].

Reliability of the DASS-21 was evaluated in the current study. It was excellent (Cronbach’s α = 0.94). The calculated validity by the critical value for Pearson’s correlation coefficient indicated the degree of freedom (df) = 185, and the critical significance value at 0.05 of the two-tailed test was 0.138. The value for Pearson’s correlation coefficient for the items was more significant than the critical value (0.138), which indicates a valid scale [25]. Additionally, to ensure the appropriateness of the measurement instruments in the current study context, content validity was assessed by obtaining the expert opinions of three professionals specializing in nursing and mental health nursing. Their feedback confirmed the relevance and clarity of the items within the International Physical Activity Questionnaire-Short Form (IPAQ-SF) and the Depression Anxiety Stress Scale-21 (DASS-21) for the target population in the study.

### 2.4. Procedure of Data Collection

After obtaining the ethical approval, a pilot study was conducted on 10% of the sample size (16 participants). However, we included 19 participants to ensure thorough testing of the feasibility and applicability of the entire data collection instrument. Thenceforth, the researcher selected the eligible students and invited them to participate in the study via email. At the beginning of the Google form, explanations were provided regarding the research objectives, implementation method, and how to complete the questionnaire for each sample. Participants were also asked to participate in the study. The research objectives and methods were presented to the participants, and written consent was obtained from them at the beginning of the Google form.

Participants completed the structured questionnaire either via Google Forms or in paper-based format.

### 2.5. Data Analysis

The completed questionnaires were extracted from the online survey and analyzed using the Statistical Package for Social Sciences (SPSS version 27). Data was presented in the form of frequency, percentage, and median. Pearson’s Chi-square test (χ^2^) and Fisher’s exact test (FET) were used to assess the association between the dependent variables.

## 3. Results

### 3.1. Participants’ Demographic Characteristics

The participants (*n* = 187) had a mean age of 20.63 ± 2.61 years. The majority were female (77%) and of Arab nationality (87.7%). Representation spanned all years of the BSN program, with 35.3% in year 1, 22.5% in year 2, 17.6% in year 3, and 24.6% in year 4.

### 3.2. PA in Participants

Table 1 represents the results of PA levels among the participants. The categorization of the PA is based on the total MET minutes/week. Data shows that 28.3% (*n* = 53) of participants were inactive, and moderate and highly active students showed 36.9% (*n* = 69) and 34.8% (*n* = 65), respectively. The MET-min/week of PA data were expressed as median. The median of vigorous activity was 340, moderate activity was 180, and walking activity was 594. (Table 1).

### 3.3. Depression, Anxiety, and Stress in Participants

**Figure 1:**
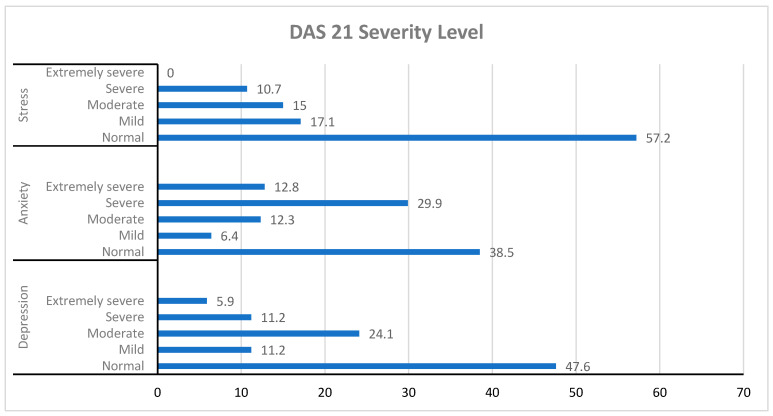
Distribution of depression, anxiety, and stress levels among nursing students (N = 187). Bars represent the proportion of students classified under each severity category according to the Depression, Anxiety, and Stress Scale-21 (DASS-21). Severity categories: depression—normal (0–9), mild (10–13), moderate (14–20), severe (21–27), extremely severe (28+); anxiety—normal (0–7), mild (8–9), moderate (10–14), severe (15–19), extremely severe (20+); stress—normal (0–14), mild (15–18), moderate (19–25), severe (26–33), extremely severe (34+).

For depression, 47.6% of students scored in the normal range, while 11.2% and 24.1% reported mild and moderate levels, respectively. Severe depression levels were reported by 11.2% of students, while extremely severe depression levels were reported by 5.9%. Regarding anxiety levels, 38.5% of students were normal, and 6.4% reported mild anxiety. However, 12.3%, 29.9%, and 12.8% of students had moderate, severe, and extremely severe anxiety levels, respectively. For stress levels, 57.2% and 17.1% of students had normal or mild stress levels. However, 15% and 10.7% of students had moderate or severe stress levels (Figure 1).

### 3.4. The Association Between the Level of PA and Demographics in Participants

A significant association was observed between physical activity and gender (χ^2^ = 9.64, *p* < 0.001). Notably, more than half of the male participants (53.5%) were involved in vigorous levels of PA. Meanwhile, most female participants (41.7%) were more engaged in moderate levels of PA. Based on nationality, Arabs have a statistically significant association with the activity level compared to non-Arab (FET = 9.09, *p* = 0.01). A large percentage of Arab students (38.4%) were involved in vigorous levels of PA compared to low or moderate activity levels. There was no significant association between PA level and the year of study in nursing (χ^2^ = 2.38, *p* = 0.89). (Table 2).

### 3.5. The Association Between Levels of Physical Activity and Depression, Anxiety, and Stress Level Among the Participants

Concerning the association between PA and depression level, among the inactive participants, 47.2% were normal, while 5.7% of inactive participants were extremely severely depressed. However, among participants who did moderate physical activity, 56.5% were normal, while 2.9% were extremely severely depressed. Moreover, 38.5% of vigorous activity participants were normal, while 9.2% of them were extremely severely depressed.

Regarding the anxiety level, less than half of inactive, moderate and vigorous participants (2.8%, 34.8%, and 70.8%) were normal. In comparison 17.0% of inactive, 15.9% of moderately active participants, and 24.6% of vigorously active participants were extremely severely anxious.

Regarding the anxiety level, the majority of vigorous participants exhibited a normal anxiety level (70.8%), compared to 34.8% in moderate participants and only 2.8% in inactive participants. Conversely, severe and extremely severe anxiety were most prevalent in the Inactive group (45.3% and 34.0%, respectively), while these categories were much less common in the moderate and vigorous groups. Mild and moderate anxiety were more frequently observed in the inactive and moderate groups, with only a few participants in the vigorous group reporting these levels.

Concerning the stress level, 62.3% of the inactive and moderately active participants were normal, and 47.7% of the vigorous active participants were normal. On the other hand, 7.5% and 4.3% of the inactive and moderate active participants were severely stressed, while 20% of the vigorous active participants were severely stressed.

Overall, there was no significant association between PA and psychological distress regarding depression (*p* > 0.05). However, a significant association was found between the PA and stress level (*p* = 0.05) and anxiety level (*p* = 0.00) (Table 3).

A multiple regression analysis was conducted to assess whether depression severity, anxiety severity, and stress severity predict physical activity category among participants. The overall regression model was statistically significant (F(3, 183) = 62.47, *p* < 0.001), explaining approximately 51% of the variance in the physical activity category (R^2^ = 0.506, adjusted R^2^ = 0.498). Anxiety severity was a significant negative predictor (B = −0.097, *p* < 0.001), indicating that higher anxiety severity is associated with lower physical activity. Stress severity was a significant positive predictor (B = 0.030, *p* < 0.001), suggesting that higher stress scores are associated with a higher physical activity category. Depression severity did not significantly predict physical activity category (B = 0.004, *p* = 0.593). Collinearity statistics (tolerance and VIF) indicated no substantial multicollinearity among the predictors (Table 4a,b).

**Figure 2:**
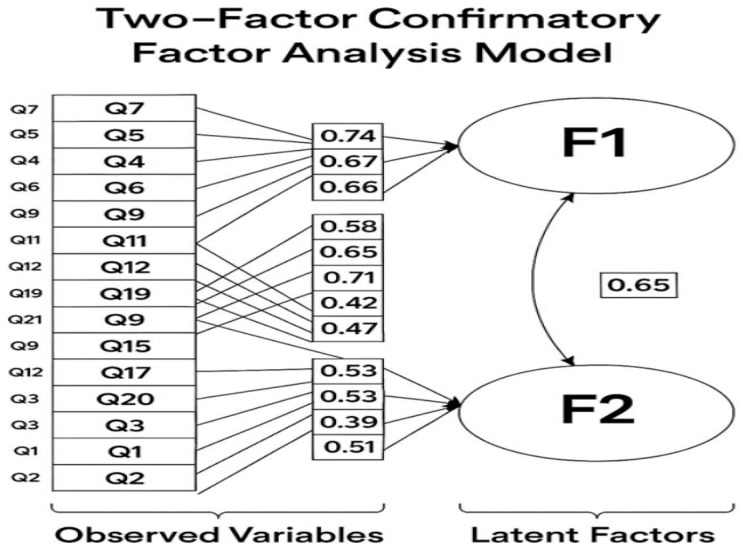
Two-factor Confirmatory Factor Analysis (CFA) model of psychological distress among nursing students.

Figure 2 illustrates a two-factor confirmatory factor analysis (CFA) model assessing the structure of psychological distress. The model identifies two latent factors: Factor 1 (F1)—physiological stress/anxiety, and Factor 2 (F2)—psychological depression/negative affect.

Factor 1 includes 14 observed items (e.g., Q4, Q5, Q7, Q8, and Q9), with standardized loadings ranging from 0.40 to 0.74. The highest loading was Q7 (“Experiencing trembling in hands”) at 0.74, indicating strong contributions from items reflecting somatic symptoms of anxiety and stress.

Factor 2 comprises seven items (e.g., Q1, Q2, Q13, and Q17), with loadings from 0.47 to 0.59, capturing emotional and cognitive symptoms such as sadness and hopelessness. Q13 showed the highest loading at 0.59. The two factors are moderately correlated (r = 0.65), indicating that physiological and psychological distress are related but distinct constructs. All factor loadings exceeded the acceptable threshold of 0.40, supporting the construct validity of the two-factor model (Figure 2).

## 4. Discussion

This study explored the relationship between PA and mental distress among the nursing students of RAKMHSU. Positive mental well-being is critical for academic success; however, psychological distress remains highly prevalent among youth in Arab nations. However, poor mental well-being due to psychological distress is predominant in the youth population of Arab nations [26]. Numerous studies have established a higher risk for distress symptoms among nursing students. Lack or misuse of PA can also adversely affect university students’ physical and mental well-being, as studies have shown a correlation between these variables. This, in turn, may be hurting the academic performance of students [27,28].

Based on the results of our study, anxiety, depression, and stress levels were reported by 67.4%, 52.4%, and 42.8% of students, respectively. Similar findings were reported by Asif et al. (2020), who showed that more than 88.4% of students have anxiety, and 75%, have depression in university students in Pakistan [29]. Along the same line, the study by Wahed and Hassan (2017) of medical school students in Egypt, found consistent with the current findings, reported a high prevalence of stress (62.4%), anxiety (64.3%), and depression (60.8%) among university students [30]. The prevalence of anxiety was 76.2%, and depression was 60.2%, respectively, among medical university students in Malaysia [31]. This consistent trend across diverse populations reinforces the universality of psychological distress among healthcare students. However, variability in reported prevalence may stem from differences in cultural stigma, access to mental health services, measurement tools, and institutional stressors. However, some studies have reported a lower percentage; Moutinho et al. (2017) [32] showed that 34.6% reported depressive symptomatology, 37.2% showed anxiety symptoms, and 47.1% stress symptoms in Brazilian medical school. The status of universities, cultural differences, and differences in the type of questionnaire may cause these differences. However, due to the prevalence of the disorders, it is necessary to emphasize the importance of the availability and provision of mental health interventions for students with mental health problems, including university counseling services. Furthermore, differences in psychological distress and PA patterns across the academic years were observed, although not statistically emphasized in this study. Senior students (e.g., third- and fourth-year) may experience elevated stress due to increased clinical exposure, academic workload, and pressure related to graduation and future employment, which aligns with findings from Chen et al. (2019) and Santangelo et al. (2019) [33,34]. In contrast, junior students may report higher anxiety due to unfamiliarity with clinical settings and academic transitions. These trends suggest the need for year-specific mental health and PA interventions tailored to the evolving stressors experienced throughout the nursing curriculum.

Male students reported higher levels of physical activity than female students, consistent with prior research in Arab countries indicating that gender strongly influences activity levels [35]. A large percentage of female students in the current research did not take part in sufficient PA. This result is consistent with the results of the study in the UAE by Dalibalta et al. (2021), where engagement in PA was higher among males than females [36]. This gender gap, corroborated across multiple Arab contexts, calls for gender-sensitive health promotion strategies, particularly addressing structural and sociocultural barriers faced by female students.

The systematic review by Chaabane et al. (2020) provides conclusive evidence of this notion, as multiple studies across Arab countries have concluded that gender discrepancy is prevalent [26]. These findings indicate that there are underlying factors that need to be explored. Sociodemographic determinants included an increase in age, marital status, urban residence, and education level, and all of these limit female participation in moderate and vigorous PA. A significant lifestyle factor identified in past research was an increase in screen time [37,38]. This may be a point of interest, as the current educational model at RAKMHSU involves a large amount of screen time, and using screen-based devices is predominant in academic and personal tasks. Given the observed screen time and inactivity correlation, future studies should explore the mediating role of digital habits in mental distress and physical inactivity, especially in health science education.

Many further factors for a gender gap in PA are found in research within the UAE by Dalibalta et al., 2021; Doyle et al., 2019 [36,39]. These factors involve cultural and social norms, where males are more encouraged to engage in PA than women; dress codes, where cultural expectations on modest dress make it challenging for women to engage in PA in public spaces; time constraints, meaning females have more expectations related to caregiving, household chores, and other work-related responsibilities; the lack of facilities where there is more access for males as opposed to females for the same sport; health status, and this is supported by data for obesity levels being higher in women than men; and the lack of awareness and education, thus leading to females not understanding the benefits of PA and how to incorporate it into daily life. These factors are context- and community-specific, and researching the impact of each factor may be the next progressive step toward increasing the PA levels for female nursing students at RAKMHSU.

Furthermore, Arab students were found to take part in more PA than non-Arab students. Research within the UAE is scarce concerning ethnicity as a contributing factor. A range of factors, such as cultural, environmental, and personal factors, influence PA. Ethnicity alone may not be a reliable predictor of PA. The high imbalance between the Arab and non-Arab populations and the imbalance in genders for the ethnicities may contribute to this study. PA can vary widely among different ethnic and cultural groups, so assuming generalizations based on ethnicity may not be appropriate. Caution is warranted when interpreting ethnicity-related trends, as PA behaviors are often more strongly influenced by social determinants, access, and institutional policies than by ethnicity alone. Further research must investigate PA access at a community rather than an ethnic level.

There is limited contemporary research performed in the UAE regarding gender discrepancy in the physical activity participation of nursing students. A meta-analysis by Yammine (2016) reports that only 35% of Arab youth participate in moderate to vigorous PA [40]. Following on from these results, the World Health Organization (WHO) reports that the UAE has significantly changed its approach to PA and positive lifestyle habits, such as by implementing the National PA Guidelines (2017) [1]. Several initiatives have also been established to promote more PA, such as the Dubai Fitness Challenge and the Abu Dhabi Community Sports Initiative. As evident in the names, these schemes are not as widespread, especially in Ras Al Khaimah, and this may be an encouraging suggestion for RAKMHSU to raise awareness and educate the students through implementing innovative schemes.

Participants engaging in vigorous physical activity tend to have significantly lower levels of anxiety, while those in the inactive group show a higher prevalence of severe and extremely severe anxiety. These findings suggest a possible association between higher levels of physical activity and lower anxiety levels.

Those who self-reported a vigorous level of PA also reported more instances of high-stress situations. These results align with the findings of the study performed by Cao et al. (2021) [41]. This is in contrast to the currently accepted findings in this field that regular PA reduces the occurrence [42]. This counterintuitive finding may indicate maladaptive use of PA as an emotional regulation tool, supporting the theory that high-intensity exercise can act as a physical stressor when not balanced with recovery time.

Vuckovic (2024) [43] proposes that the physiological and psychological mechanism refers to releasing endorphins that elevate the mood by reducing feelings of distress. Following this, there is a reduction in cortisol associated with this, as cortisol increases stress levels [43]. As suggested by the research, this mechanism may be misbalanced. Students who feel stressed may overuse PA as a coping mechanism. This consistent and long-term increase, along with the cortisol increase due to academic pressure without sufficient time to recover, results in unhealthy and long-term feelings of stress. This explains why those who reported consistent and vigorous active PA felt more instances of stress [41]. Vigorous PA was shown to be associated with higher perceived stress levels.

These findings align with the concept of ‘exercise as a stressor,’ wherein physical activity itself imposes physiological strain that can amplify perceived stress if not balanced with recovery [44]. Research on this notion is non-existent within the UAE. It is essential to acknowledge that exercise itself imposes stress on the body. This physical stress may be interacting with the other sources of stress within a student’s life, leading to a cumulative effect of stress from multiple sources [44]. The key finding in this research is the application of the appropriate mode of PA. To counteract this issue, students are encouraged to use PA as a distraction and a relaxation technique to avoid the stressors while using less intense stress relief methods, such as pursuing low-intensity PA hobbies [45,46]. Individual differences and contextual factors must be emphasized to examine the effect of stress, and this may be performed through personalized approaches based on stress levels, coping capability, and appropriate PA modalities.

Research agrees that common factors that may be contributing to this were reported as disinterest in the course, low GPA, concerns about future placements, workload, fear of unknown circumstances, mistakes during clinical practices, and working under pressure [33,34]. Risk factors for nursing students include sociodemographic characteristics such as age, year of study, family relationship crisis, financial difficulties, self-perceived mental health, a lack of leisure and quiet time, sleep problems, and work-related factors such as clinical specialty.

Students must be encouraged to take part in sufficient PA but also engage in low-intensity activities such as listening to music, spending time outdoors, reading, and gardening as a coping mechanism for stressors, both academic and lifestyle, to prevent burnout, which is high among the nursing student population [45].

PA data differentiating nationals and non-nationals in the Gulf Cooperation Council (GCC) countries are rare. They must be explored by future research, as a significant portion of the population is non-nationals. Furthermore, demographic specificities in future studies may be key for developing, implementing, and monitoring interventions.

The findings of this study emphasize the need for early identification and targeted intervention strategies addressing psychological distress in nursing students. Integrating structured PA programs and wellness workshops into the nursing curriculum can not only enhance students’ coping mechanisms but may also reduce future burnout risk in clinical settings. Nurse educators and clinical supervisors should adopt a proactive role in modeling and encouraging stress management techniques and physical wellness habits, ultimately supporting a more resilient future nursing workforce.

The confirmatory factor analysis (CFA) provided empirical support for a two-factor structure representing distinct but interrelated dimensions of psychological distress: physiological stress/anxiety and psychological depression/negative affect. The strong factor loadings for items such as trembling, restlessness, and somatic discomfort under Factor 1, and emotional symptoms like sadness and hopelessness under Factor 2, align with theoretical expectations and the existing literature on stress and mood disorders. The moderate correlation between the two factors (r = 0.65) suggests that while these domains are conceptually separate, they frequently co-occur, reflecting the multidimensional nature of psychological distress. This validated structure not only enhances the construct validity of the tool but also reinforces the importance of addressing both somatic and emotional symptoms in clinical and educational interventions targeting mental health among university students. The CFA results validate the bidimensional structure of psychological distress and support the tool’s cross-cultural applicability among nursing students in the UAE context. This structural clarity can aid in designing targeted screening and intervention tools.

### Strengths and Limitations 

The current study examines the relation between physical activity and psychological distress among nursing students in the UAE, a population recognized as being at high risk for mental health challenges. It highlights the importance of developing strategies and programs that increase nursing students’ awareness of the benefits of physical activity in reducing psychological distress and enhancing their overall health and well-being.

This study presented the characteristic limitations of single-site, cross-sectional studies, i.e., it did not assess cause and effect. The sample size was small, which limits the generalizability of the findings. A self-administered questionnaire was used; hence, respondents’ bias cannot be ruled out. A deeper analysis was not carried out through interviews and focus groups.

## 5. Conclusions

The study reveals a clear gender gap in physical activity: male students engaged in more moderate to vigorous activity than female students. Ethnicity was also found to be significantly associated with the level of PA. However, the transferability of this data for future interpretation must be performed with caution as a range of cultural and religious factors are responsible for the level of PA rather than just ethnicity alone. Implementing locally informed and evidence-based strategies to promote PA for nursing students, especially with the female population, and addressing the barriers to PA should be the primary aim of future studies. Therefore, future studies must explore specific factors within RAKMHSU that may influence female PA participation. Future studies must also explore specific cultural factors that may be causing the inconsistency in PA participation among nationalities. Future studies should incorporate qualitative approaches, such as interviews or focus groups, to explore students’ lived experiences and perceptions of physical activity and psychological distress. This mixed-methods approach would provide richer, more comprehensive insights to complement quantitative findings.

### Relevance to Clinical Practice

Sustaining student mental health requires early awareness and preventive strategies. Equipping nursing students with the skills to recognize and manage stressors is essential for enhancing quality of life, academic success, and long-term career sustainability.

## Figures and Tables

**Table 1:** Levels of physical activity among nursing students (N = 187).

**Physical Activity Level**		
**Items**	**Number (*n =* 187)**	**Percent (%)**
Inactive	53	28.3
Moderate	69	36.9
Vigorous	65	34.8
**Median of Physical activity among the participants (min/week),**		
Vigorous MET-min/wk	340.00 min/wk	
Moderate MET-min/wk	180.00 min/wk	
Walking MET-min/wk	594.00 min/wk	
**Total MET-min/wk**	**1497.00 min/wk**	

Note: MET = Metabolic Equivalent of Task. PA levels were categorized using IPAQ-SF guidelines.

**Table 2:** Association between physical activity levels and demographic characteristics of participants (N = 187).

*Variable*	Inactive *n* (%)	Moderate *n* (%)	Vigorous *n* (%)	Test Statistic	*p*-Value
***Gender***				χ^2^ = 9.64	<0.001 *
*Male* (*n* = 43)	11 (25.6)	9 (20.9)	23 (53.5)		
*Female* (*n* = 144)	42 (29.2)	60 (41.7)	42 (29.2)		
***Nationality***				FET = 9.09	0.010 *
*Arab* (*n =* 164)	43 (26.2)	58 (35.4)	63 (38.4)		
*Non-Arab* (*n* = 23)	10 (43.5)	11 (47.8)	2 (8.7)		
***Year of Study***				χ^2^ = 2.38	0.890
*Year 1* (*n* = 66)	19 (28.9)	25 (37.9)	22 (33.3)		
*Year 2* (*n =* 42)	11 (26.2)	16 (38.1)	15 (35.7)		
*Year 3* (*n =* 33)	12 (36.4)	12 (36.4)	9 (27.3)		
*Year 4* (*n* = 46)	11 (23.9)	16 (34.8)	19 (41.3)		

Note: χ^2^ = Chi-square test; FET = Fisher’s exact test; * *p* ≤ 0.05 considered statistically significant.

**Table 3:** Association between physical activity levels and psychological distress (N = 187).

		Inactive 53 (28.3%)	Moderate 69 (36.9%)	Vigorous 65 (34.8%)	Test Statistic	*p*-Value
		*n* (%)	*n* (%)	*n* (%)		
**Depression Level** **Mean = 10.59, SD = 9.28**	Normal	25 (47.2%)	39 (56.5%)	25 (38.5%)	χ^2^ = 6.92	0.56
	Mild	7 (13.2%)	6 (8.7%)	8 (12.3%)		
	Moderate	14 (26.4%)	14 (20.3%)	17 (26.2%)		
	Severe	4 (7.5%)	8 (11.6%)	9 (13.8%)		
	Extremely severe	3 (5.7%)	2 (2.9%)	6 (9.2%)		
**Anxiety Level** Mean = 9.58, SD = 5.92	Normal	2 (2.8%)	24 (34.8%)	45 (70.8%)	FET = 99.34	0.00 *
	Mild	7 (13.2%)	4 (5.8%)	1 (1.5%)		
	Moderate	2 (3.8%)	8 (11.6%)	13 (20.0%)		
	Severe	24 (45.3%)	27 (39.1%)	5 (7.7%)		
	Extremely severe	18 (34.0%)	6 (8.7%)	0 (0.0%)		
**Stress Level** Mean = 12.32, SD = 9.23	Normal	33 (62.3%)	43 (62.3%)	31 (47.7%)	χ^2^ = 12.40	0.05 *
	Mild	11 (20.8%)	12 (17.4%)	9 (13.8%)		
	Moderate	5 (9.4%)	11 (15.9%)	12 (18.5%)		
	Severe	4 (7.5%)	3 (4.3%)	13 (20.0%)		

χ^2^ = Chi-square test; FET = Fisher’s exact test; * significant difference at *p* level ≤ 0.05.

**Table 4:** (**a**) Multivariate linear regression predicting physical activity level (N = 187). (**b**) Regression coefficients for predictors of physical activity.

(**a**)											
**Outcome Variable**	**R**	**R Square**	**Adjusted R Square**			**Std. Error of the Estimate**			**F**	***p*-Value**	
Physical Activity Level	0.711	0.506	0.498			0.563			62.465	0.000	
(**b**)											
**Predictor**	**B**	**Std. Error**	**Beta**	**t**	***p*-value**		**95% CI (Lower, Upper)**	**Tolerance**			**VIF**
Constant	2.579	0.087		29.548	0.000		2.407, 2.752				
Anxiety Score	−0.097	0.007	−0.720	−13.152	0.000		−0.111, −0.082	0.902			1.109
Stress Score	0.030	0.008	0.343	3.643	0.000		0.014, 0.045	0.305			3.283
Depression Score	0.004	0.008	0.051	0.535	0.593		−0.012, 0.020	0.299			3.348

(a) Note: Dependent variable = physical activity level; predictors = anxiety, stress, and depression scores. Model significant at *p* < 0.001. (b) Note: Dependent variable = physical activity level. *p* ≤ 0.05 considered statistically significant. No multicollinearity detected (all VIF < 4).

## Data Availability

Data is contained within the article. The original contributions presented in this study are included in the article. Further inquiries can be directed to the corresponding author.
